# Shadows of trauma: an umbrella review of the prevalence and risk factors of post-traumatic stress disorder in children and adolescents

**DOI:** 10.1186/s13034-025-00879-4

**Published:** 2025-04-29

**Authors:** Tadesse Tarik Tamir, Berhan Tekeba, Enyew Getaneh Mekonen, Deresse Abebe Gebrehana, Alebachew Ferede Zegeye

**Affiliations:** 1 Department of Pediatrics and Child Health Nursing, School of Nursing, College of Medicine and Health Sciences, University of Gondar, Gondar, Ethiopia; 2 Department of Surgical Nursing, School of Nursing, College of Medicine and Health Sciences, University of Gondar, Gondar, Ethiopia; 3 Department of Internal Medicine, School of Medicine, College of Medicine and Health Sciences, University of Gondar, Gondar, Ethiopia; 4 Department of Medical Nursing, School of Nursing, College of Medicine and Health Sciences, University of Gondar, Gondar, Ethiopia

**Keywords:** Prevalence, Factors, Post-traumatic stress, Umbrella review, Children, Adolescents

## Abstract

**Introduction:**

Post-Traumatic Stress Disorder (PTSD) is a significant mental health concern affecting children and adolescents, often resulting from exposure to traumatic events such as violence, natural disasters, or abuse. A substantial number of children and adolescents experience these traumatic events; however, the reported prevalence of PTSD in this population varies widely across systematic reviews and meta-analyses. This umbrella review aims to synthesize findings from multiple systematic reviews and meta-analyses to provide a comprehensive estimate of PTSD prevalence and identify key risk factors associated with the disorder.

**Methods:**

A comprehensive literature search was conducted across several databases, including PubMed, Scopus, EMBASE, and others, using the COCOPOP framework. Systematic reviews and meta-analyses published between January 1, 2014, and December 1, 2024, were included. Data were extracted by two reviewers independently and analyzed using Stata 17 with a random-effects meta-analysis model.

**Results:**

A total of twelve studies were included, with a combined sample size of 121,333 participants. The pooled prevalence estimate for PTSD among children and adolescents was found to be 25% (95% CI: 20-30%), with substantial heterogeneity (I² = 99.9%). Subgroup analyses indicated variations in prevalence based on publication year and the number of primary studies included in the systematic reviews. Key risk factors identified included older age, female gender, low social support, feelings of entrapment, and experiencing bereavement.

**Conclusions:**

The prevalence of PTSD among children and adolescents exposed to trauma is notably high. Key contributing factors include older age, female gender, low social support, feelings of entrapment, and experiencing bereavement. Targeted interventions focusing on these risk factors, such as enhancing social support systems and providing early mental health interventions, are essential to improve outcomes for this vulnerable population. Further research is needed to refine these strategies and ensure they effectively meet the needs of children and adolescents affected by trauma.

**Supplementary Information:**

The online version contains supplementary material available at 10.1186/s13034-025-00879-4.

## Introduction

Post-Traumatic Stress Disorder (PTSD) is a significant mental health concern that affects children and adolescents, often resulting from exposure to traumatic events such as violence, natural disasters, or abuse [1–3]. The impact of PTSD during these formative years can be profound, influencing not only psychological well-being but also physical health, social relationships, and academic performance [4]. Understanding the prevalence, risk factors, and treatment options for PTSD in younger populations is crucial for developing effective interventions and support systems [5].

While much of the research on PTSD has historically focused on adults, particularly military veterans [6, 7], there is a growing recognition of the profound impact that trauma can have on children and adolescents [8]. This vulnerable population is exposed to a wide range of traumatic experiences, including natural disasters, accidents, abuse, and violence, which can lead to the development of PTSD [8]. Children and adolescents are particularly vulnerable to traumatic events due to their limited coping strategies and reduced ability to protect themselves effectively [9].

Studies have shown that a significant proportion of children and adolescents experience traumatic events, with varying rates of PTSD development depending on the type and severity of the trauma [8, 10]. Systematic reviews and meta-analyses indicate that the prevalence of PTSD in this population ranges from 15 to 35% following traumatic events [11, 12]. Primary studies have linked PTSD to several factors, including but not limited to age, gender, location, education level, the cause of trauma, personal or family history of mental illness, and social support [13, 14]. Understanding the comprehensive prevalence and risk factors of PTSD in children and adolescents is crucial for developing effective prevention and intervention strategies.

PTSD is a significant mental health concern among children and adolescents, with profound implications for their development and well-being [15–17]. While numerous systematic reviews and meta-analyses (SRMAs) have examined the prevalence and risk factors of PTSD in this population, inconsistencies in their findings have created a fragmented understanding of the issue [11, 12]. These inconsistencies stem from variations in methodologies, populations, and contexts, leaving a critical gap in the literature: a comprehensive synthesis of pooled prevalence estimates through an umbrella review approach. This gap limits our ability to provide a unified understanding of the global burden of PTSD in young individuals and identify consistent risk factors across diverse contexts.

This umbrella review aims to address this gap by synthesizing findings from multiple SRMAs to provide a robust, comprehensive estimate of PTSD prevalence in children and adolescents. Additionally, it seeks to identify the key risk factors associated with PTSD, thereby highlighting areas where targeted support and resources are most needed. By integrating evidence across studies, this review not only fills a critical void in the research landscape but also enhances the precision and reliability of prevalence estimates. The findings from this study are intended to inform clinicians, policymakers, and researchers, guiding efforts to improve mental health outcomes for children and adolescents affected by trauma. By underscoring the importance of addressing mental health issues in young individuals, this review contributes to the broader goal of fostering resilience and recovery in the face of trauma.

## Methods and materials

### Study protocol

The protocol for this umbrella review was developed in compliance with the Preferred Reporting Items for Systematic Reviews and Meta-Analysis Protocols statement. Initially, the presence of an identical umbrella review was checked on PROSPERO, which showed that no similar studies had been submitted. Then, the study’s protocol was established and registered (CRD42024620034).

### Searching strategy

An inclusive literature search was conducted on systematic reviews and meta-analyses related to Post-Traumatic Stress Disorder and its associated factors using the COCOPOP framework across several databases, including PubMed, Scopus, EMBASE, Science Direct and search engines Google Scholar and Google. Publications were selected from previous studies that met the eligibility criteria. A search strategy was developed for the databases by combining key terms using Boolean operators (“AND” and “OR”). We included all systematic reviews and meta-analyses published between January 1, 2014, and December 1, 2024. Additionally, snowball sampling techniques were employed to identify further research from the citation lists of the papers found in the databases.

To identify relevant studies for our umbrella review on the prevalence and associated factors of PTSD in children and adolescents, we developed a comprehensive search strategy that incorporated a variety of terms related to PTSD and its synonyms. Our search terms were categorized into four main groups: disorder terms, outcome terms, population terms, and study type. For disorder terms, we included variations such as “Acute Post-Traumatic Stress Disorder,” “Chronic Post-Traumatic Stress Disorder,” “Delayed Onset Post-Traumatic Stress Disorder,” “Moral Injury,” “Neuroses, Post-Traumatic,” and “PTSD,” among others, to capture all manifestations of the disorder. To focus on our specific interests, we added outcome terms such as “Prevalence,” Magnitude,” “Epidemiology,” “Incidence,” “Risk Factors,” “Predictors,” “Determinants,” “Contributing Factors” and “Associated Factors.” We targeted studies involving children and adolescents by using population terms like " “Pediatric”, “Kids”, “Child”, “Children”, “Adolescents”, and “Young” Lastly, to ensure the inclusion of high-quality evidence, we limited our search to systematic reviews and meta-analyses, utilizing terms such as “Systematic Review,” “Meta-analysis,” and “Systematic Review and Meta-analysis.” The final search strategy combined these terms using Boolean operators, structured as follows:

(“Acute Post-Traumatic Stress Disorder” OR “Chronic Post-Traumatic Stress Disorder” OR “Delayed Onset Post-Traumatic Stress Disorder” OR “Moral Injury” OR “Neuroses, Post-Traumatic” OR “PTSD” OR “Post-Traumatic Stress Disorder” OR “Post-Traumatic Stress Disorders” OR “Stress Disorder, Post-Traumatic”) AND (Prevalence OR Magnitude OR Epidemiology OR Incidence OR “Risk Factors” OR Predictors OR Determinants OR “Contributing Factors” OR “Associated Factors”) AND (Pediatric OR Kids OR Child OR Children OR Adolescents OR Young) AND (“Systematic Review” OR “Meta-analysis”).

### Eligibility criteria

This review encompasses systemic reviews and meta-analyses of studies published in English that focus on the prevalence and risk factors of PTSD among children and adolescents. To ensure the timeliness of the included studies, as it is widely recommended that SRMAs published within the past ten years are less likely to be outdated and more likely to reflect the most current evidence and methodologies in the field, only studies published on or after January 1, 2014, were included in this umbrella review.

Articles that did not measure the outcome of interest were excluded, as well as narrative reviews, primary studies, qualitative reviews, expert opinions, case reports, editorials, correspondence, and methodological studies. This approach allows us to maintain a clear focus on robust evidence regarding PTSD in the specified population.

### Study selection

Based on the inclusion and exclusion criteria, all searched studies were transferred to Endnote x9, a reference management software, to discard duplicate studies. After duplications were removed, two reviewers independently read the titles and abstracts of the remaining articles to identify both potentially eligible articles and any articles for which a determination could not be made from the title and abstract alone. Then, the selected full text of the remaining articles was examined for eligibility.

### Outcomes of interest

The primary objective of this study was to determine the overall prevalence of PTSD among children and adolescents. The second objective of this umbrella review was to identify risk factors of among children and adolescents, which were evaluated using adjusted odds ratios/relative risk/beta coefficients from prior SRMA studies.

### Data extraction and management

Two authors (TTT and BT) carried out data extraction from the included SRMA studies using a standardized data abstraction form, developed in excel spreadsheet. Articles were screened and selected first based on their title and abstract, and then the full-text was reviewed. In cases of dispute, discussions with additional reviewers were held to determine the final article selection to include in this umbrella review. Following the comprehensive searching, possibly eligible publications were imported into EndNote. Duplicate studies were deleted in cases where two or more papers shared similar features. Structured data extraction in a Microsoft Excel spreadsheet was designed and implemented.

### Quality assessment

The quality of the included article will be evaluated by two separate reviewers using the Assessment of Multiple Systematic Reviews (AMSTAR 2) method. The new quality assessment tool builds on the previous AMSTAR. The specific included article was extracted based on the 16 times of the AMSTAR 2 tool. The system categorizes specific SMR evidence into four categories based on its quality: high, moderate, low, and critically low [18] (Table 1).

**Table 1:** The characteristics of the included systematic reviews and metanalyses

Study	Year	Traumatic events	Setting	N*0* of 1^0^ studies	Disease	Total sample	Prevalence	Quality
Tamir et al. [12]	2024	General Trauma	Africa	17	3215	8,930	0.360	Moderate
Alizadeh et al. [20]	2023	COVID 19	Global	43	75,707	329,159	0.230	Moderate
O-Etxebarria et al. [21]	2023	COVID 19	Asia	6	10,188	72,774	0.140	Moderate
Kanan et al. [22]	2022	Conflict	Syria	26	4104	11,400	0.360	Moderate
Yang et al. [23]	2022	COVID 19	China, USA, Italy	10	4894	17,385	0.282	Moderate
Agbaria et al. [24]	2021	Political violence	Palestine	28	5444	15,121	0.360	Moderate
Woolgar et al. [25]	2021	General Trauma	Woolgar	18	417	1941	0.215	Moderate
Turgoose et al. [26]	2021	Paediatric surgery	Turgoose	16	187	1169	0.160	Moderate
Latuperissa et al. [27]	2020	Earthquake	NR	14	12,843	33,620	0.382	low
Dai et al. [28]	2018	RTA	NR	11	306	1532	0.200	low
Tang et al. [11]	2017	Earthquake	NR	15	3461	22,931	0.151	Moderate
Alisic et al. [29]	2014	General Trauma	Global	43	567	3563	0.159	low

*O-Etxebarria*: Ozamiz-Etxebarria, *NR* Not reported, *RTA* road traffic accident

### Data synthesis and statistical analysis

Data were extracted using Microsoft Excel spreadsheet and exported to the STATA 17 statistical software, where all statistical data analyses were carried out. The extracted data were displayed using texts, tables, and forest plots. The standard error of prevalence for each SRMA study was analyzed using the binomial distribution. The combined prevalence of the SRMA studies was checked for heterogeneity using I-square (I^2^) test. Heterogeneity across the included studies was classified as low, moderate, or high when the values of I-square were < 25%, 50-75%, and 75%, respectively [19].

A random-effects meta-analysis model with Der Simonian and Laird’s method was used to determine the pooled prevalence of PTSD. Sub-group analysis was study characteristics to identify potential causes of heterogeneity among studies. In addition, we have executed a leave-one-out sensitivity analysis to determine the effect of single SRMA studies on the pooled estimate. Then, forest plots and tables were used to display the pooled estimates with their corresponding 95% confidence intervals. Statistically, publication bias or small study effect was assessed using Egger’s test with p-value less than 0.05 used to declare the significance of publication bias. Besides, publication bias was detected using Funnel plot symmetry.

## Results

### Selection of systematic reviews and meta-analyses

**Fig. 1:**
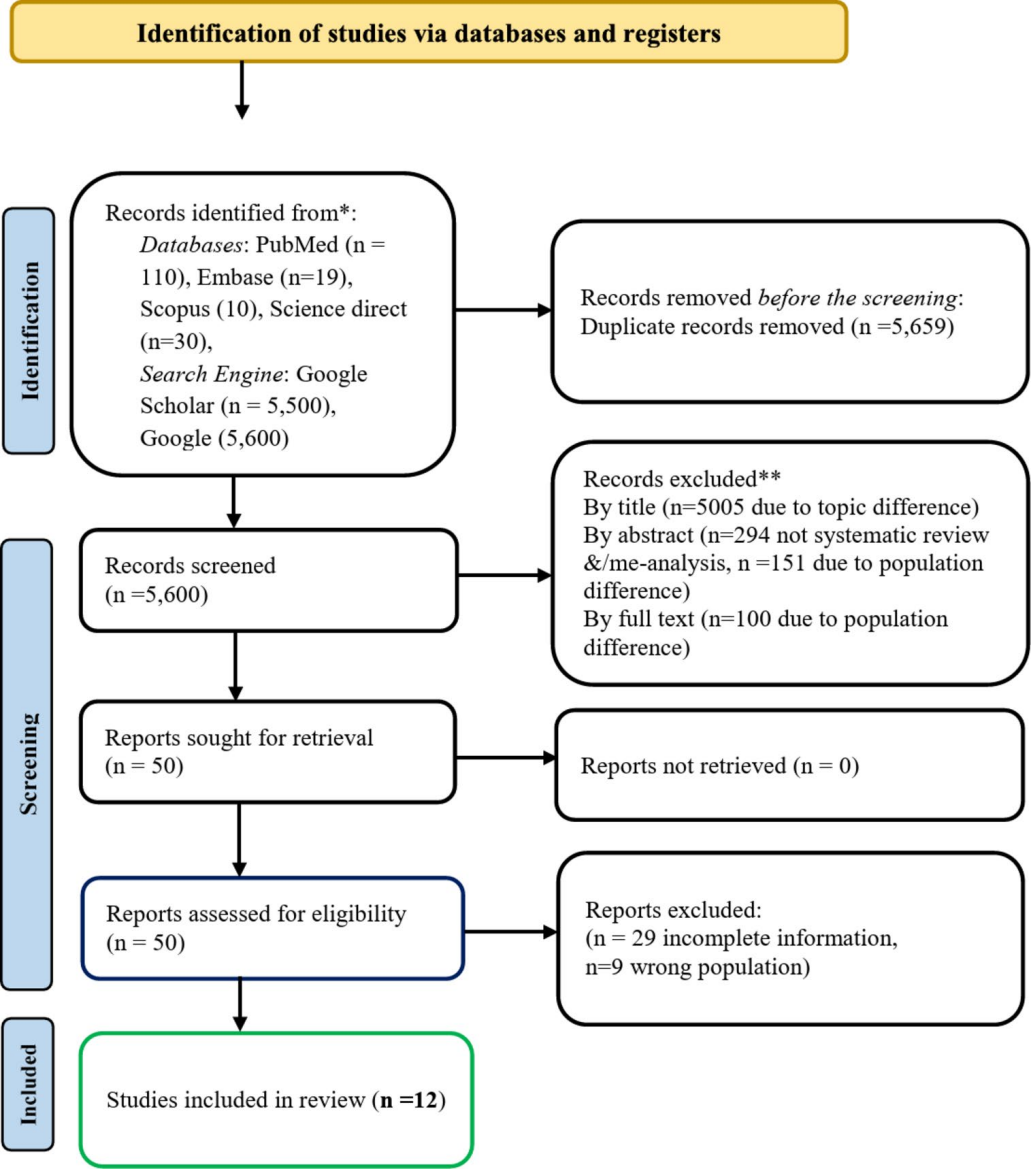
PRISMA 2020 flow diagram for searches of databases and registers

In the systematic selection of studies for this Umbrella review, a comprehensive search strategy was employed across multiple databases, resulting in the identification of a total of 110 records from PubMed, 10 from Scopus, 20 from Science Direct, extensive 5,500 from Google Scholar, and 5,600 from Google. This strategic approach ensured a wide coverage of relevant literature. Following the identification phase, duplicate records were removed to streamline the dataset, leading to a refined pool of unique studies for further evaluation. The screening process involved a thorough examination of the titles, which resulted in the exclusion of 505 records that did not meet the criteria for systematic reviews, as they focused primarily on population differences. Importantly, no records were excluded due to retrieval issues. Subsequent eligibility assessments revealed that 29 reports lacked complete information and 9 reports were of the wrong population and were 38 in total, excluded from further consideration. Ultimately, 12 studies were included in the review, consisting of relevant systematic reviews and meta-analyses that adhered to the predefined inclusion criteria (Fig. 1). This methodical selection process underscores the rigorous approach taken to ensure the inclusion of high-quality evidence, thereby enhancing the credibility and comprehensiveness of the findings presented in this umbrella review.

### Characteristics of SRMAs inluded in the umbrella review

This umbrella review encompasses systematic reviews and meta-analyses published between 2014 and 2024, focusing on various traumatic events and their association with the prevalence of PTSD among children and adolescents. The studies included cover a range of traumatic experiences, including general trauma, COVID-19, conflict, political violence, and natural disasters such as earthquakes.

In total, 12 studies were analyzed, with a combined sample size exceeding 121,333 participants. The prevalence rates of PTSD varied across the studies, reflecting the diverse contexts and traumatic events examined. The quality of the studies, assessed using a AMASTER 2, ranged from low to moderate. Notable studies include those by Alizadeh et al. (2021) and Ozamiz-Etxebarria et al. (2023), which reported the highest total sample sizes of 329,159 and 72,774, respectively, highlighting the significant impact of the COVID-19 pandemic on mental health outcomes in children and adolescents (Table 1).

### Prevalence of PTSD in children and adolescents

**Fig. 2:**
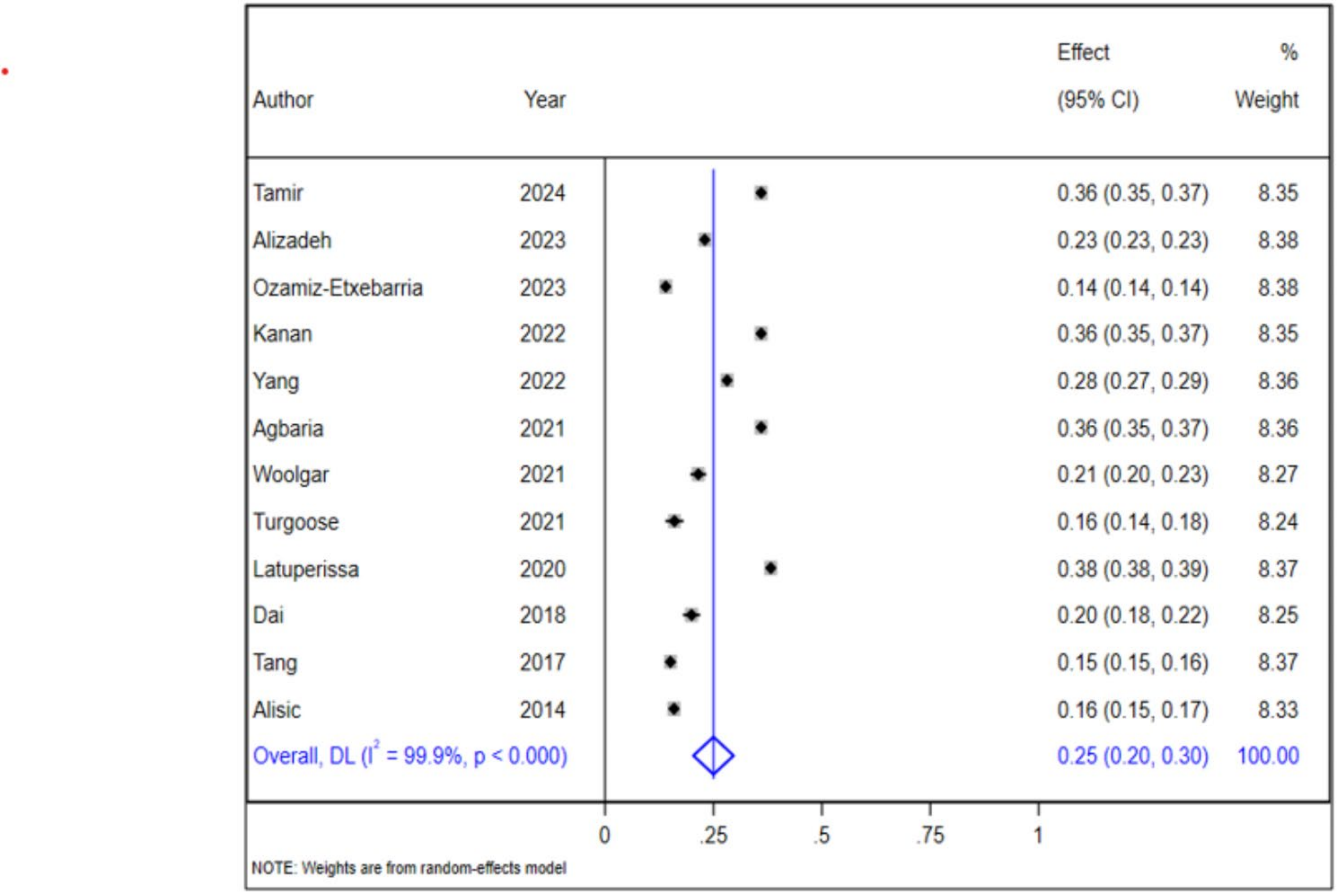
Pooled prevalence of PTSD among Children and Adolescents

In this umbrella review encompassing multiple systematic reviews and meta-analyses, we examined the prevalence of post-traumatic stress disorder (PTSD) among children and adolescents. The pooled prevalence estimate for PTSD was found to be 25%, with a 95% confidence interval (CI) ranging from 20 to 30%. Importantly, we observed substantial heterogeneity across the studies, which necessitated the application of a random-effects model for our analysis. The I² statistic revealed an exceptional degree of variability attributed to heterogeneity, measuring at 99.9% (*p* < 0.001). For further insights into these findings, refer to Fig. 2 below, which illustrates the results visually.

### Subgroup analysis of the prevalence of PTSD in children and adolescents

**Fig. 3:**
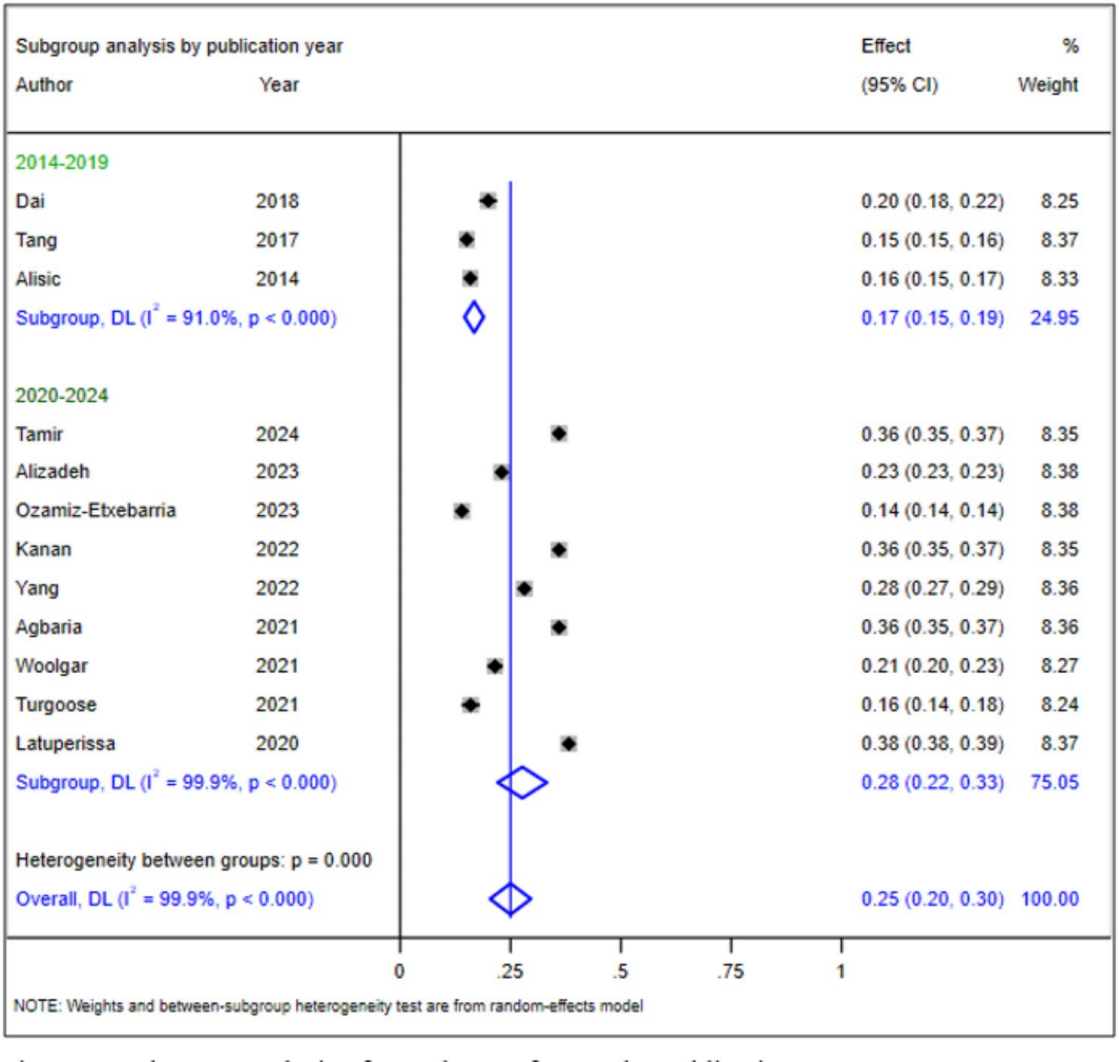
Subgroup analysis of prevalence of PTSD by publication year

**Fig. 4:**
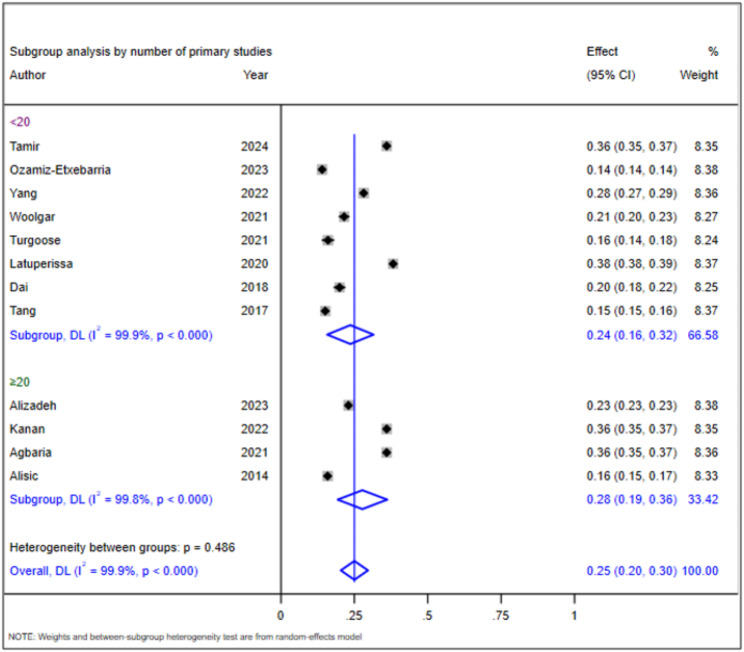
Subgroup analysis of prevalence of PTSD by number of primary studies

The observed heterogeneity prompted us to conduct subgroup analyses to identify its source. We performed these analyses based on the year of publication and the number of primary studies included in the SRMA. The prevalence of PTSD was 17% (95% CI: 15–19%) with an I² of 91.0% (*p* < 0.001) for SRMAs published more than five years ago and 28% (95% CI: 22–33%) with an I² of 99.9% for SRMAs published within the past five years (*p* < 0.001) (Fig. 3). For SRMAs that assessed PTSD using fewer than 20 primary studies, the prevalence was 24% (95% CI: 16–32%) with an I² of 99.9% (*p* < 0.001), compared to 28% (95% CI: 19–36%) with an I² of 99.8% for SRMAs including 20 or more studies (Fig. 4). This demonstrates the persistence of heterogeneity even after subgroup analysis, highlighting the need for further sensitivity analysis.

### Sensitivity analysis of the prevalence of PTSD in children and adolescents

**Fig. 5:**
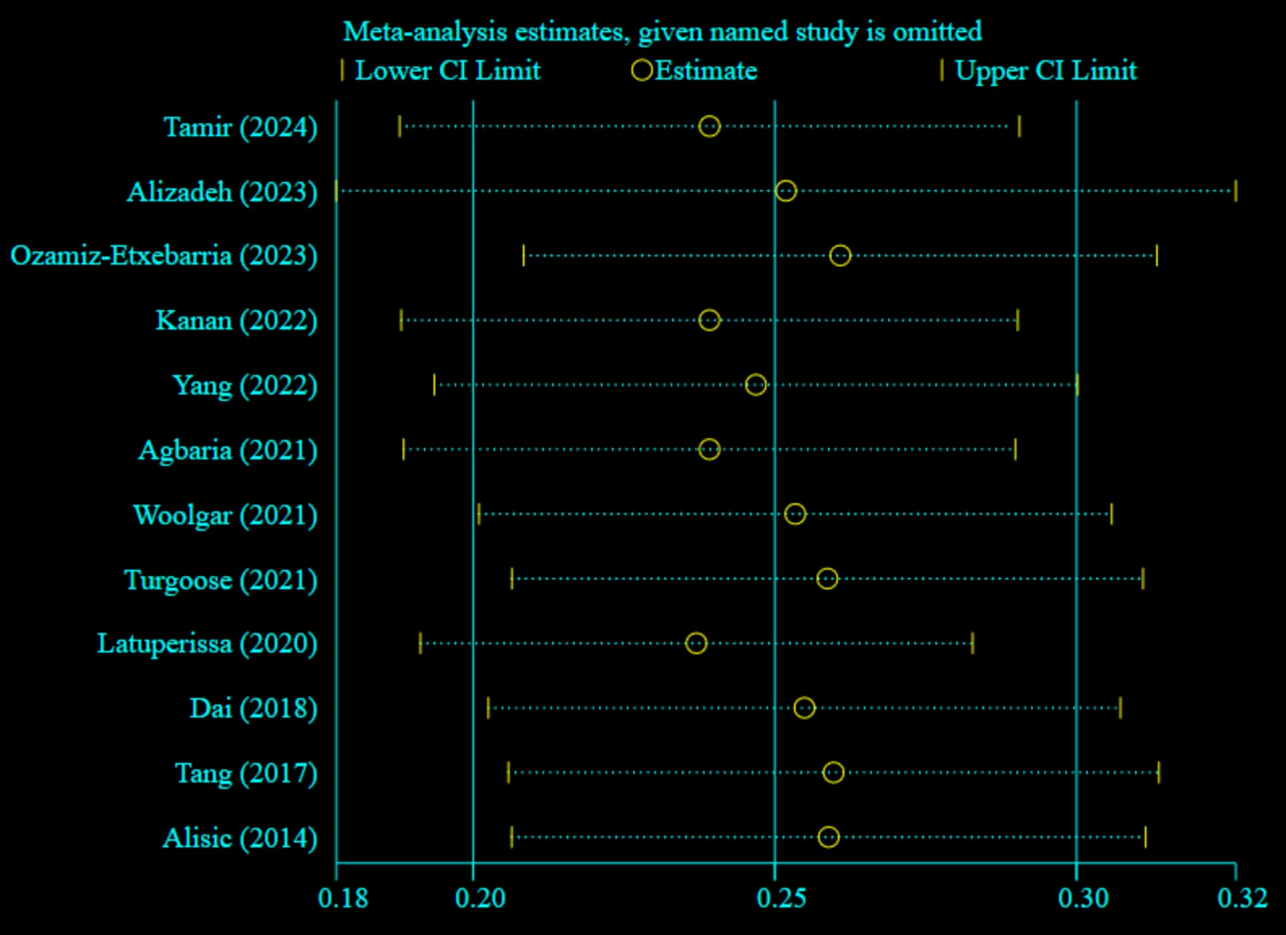
Sensitivity analysis of prevalence of PTSD among children and adolescents

The sensitivity analysis was conducted to assess the heterogeneity of the included studies by systematically excluding one study at a time. This approach allowed us to evaluate the impact of each individual study on the overall prevalence of PTSD reported in our review. The results indicated that all values fell within the estimated 95% confidence interval (CI), suggesting that the omission of any single study did not significantly alter the overall prevalence reported in this systematic review and meta-analysis (as shown in Fig. 5).

### Test of publication bias of the prevalence of PTSD in children and adolescents

**Fig. 6:**
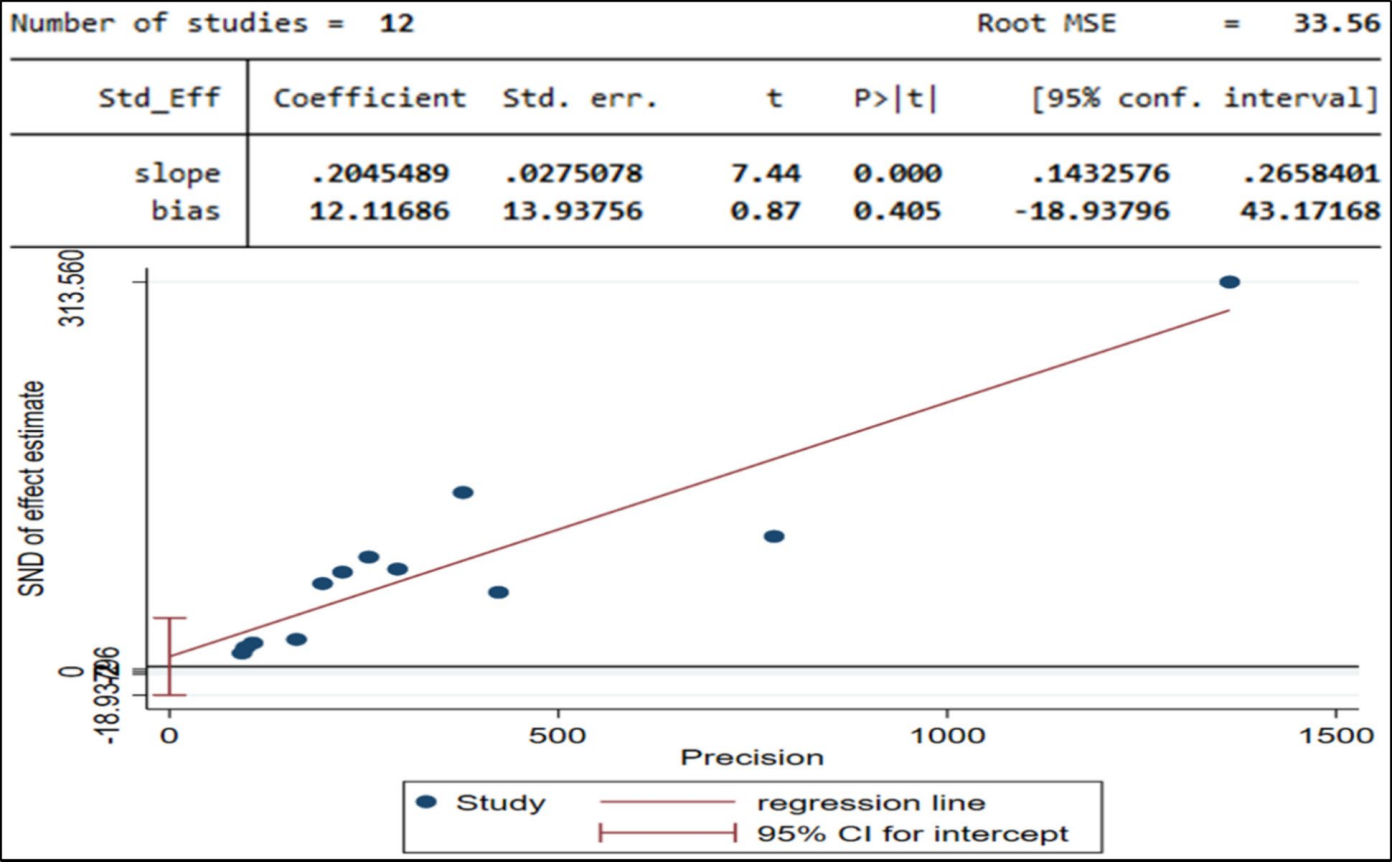
Egger test of small-study effects of prevalence of PTSD among children and adolescents

Small-study effects describe the tendency for smaller studies to report more favorable intervention effects. The estimated bias coefficient (intercept) is 12.11686, with a standard error of 13.93756 This standard error indicates the level of uncertainty in our estimate of the bias coefficient. A larger standard error implies greater uncertainty, while a smaller one suggests more precise estimates. The p-value for the bias coefficient is 0.87. This p-value helps determine the statistical significance of the bias coefficient. In this case, a p-value above 0.05 means the bias coefficient is not statistically significant. Therefore, the test results do not provide strong evidence of small-study effects (Fig. 6). Essentially, this suggests that the bias from selectively publishing positive findings is not prominent in this instance.

### Risk factors of PTSD in children and adolescents

In conducting this umbrella review, a comprehensive search was performed to identify systematic reviews and meta-analyses (SRMAs) that examined both the prevalence and associated risk factors of PTSD in children and adolescents. While a sufficient number of SRMAs were found to facilitate a robust meta-analysis of the prevalence of PTSD, the search yielded only two SRMAs specifically addressing the associated risk factors.

This limited availability of SRMAs on risk factors presented a significant challenge in synthesizing a comprehensive meta-analysis for this section. The scarcity of high-quality, aggregated data on the determinants of PTSD in children and adolescents underscored a critical gap in the existing literature. Consequently, a systematic synthesis of the available evidence was performed, supplemented by high-quality primary studies where necessary, to provide insights into the risk factors associated with PTSD in this vulnerable population.

We observed that older age, high educational attainment, feelings of entrapment, experiencing fear, injury, or bereavement, and witnessing injury or death during the traumatic event were reported as significant determinants of PTSD in children by one of the SRMAs included in this review [11]. Additionally, another SRMA published in 2024, which was eligible for and included in this umbrella review, highlighted that being older (above 14 years) and experiencing bereavement (the death of family members or loved ones) were risk factors for PTSD in the pediatric population [12]. Moreover, female gender, proximity to a trauma event, preexisting psychiatric disorders, parental psychopathology, low social support, duration of trauma, severity of pain from trauma, and comorbid mental illness were consistently identified as risk factors for PTSD in several primary studies [9, 30–33].

## Discussion

The prevalence of post-traumatic stress disorder and its risk factors among children and adolescents is a critical area of concern, given the profound impact trauma can have on young individuals’ mental health and development.

This umbrella review pooled findings from multiple systematic reviews and meta-analyses to provide a comprehensive estimate of PTSD prevalence in this population. Our analysis reveals a pooled prevalence estimate of 25% (95% CI: 20-30%), indicating that a significant proportion of children and adolescents exposed to trauma develop PTSD. This finding is significant as it aligns with existing evidence indicating that approximately 15–30% of veterans experience PTSD following traumatic events [34]. Such a comparison underscores the substantial psychological impact of trauma across different populations, suggesting that children and adolescents may be at a similarly high risk for developing PTSD as veterans.

However, the prevalence identified in this review exceeds the estimates provided by the National Centers for PTSD, which reported that between 6% and 15% of children and adolescents exposed to trauma develop PTSD [9]. This discrepancy raises important questions regarding the methodological approaches and diagnostic criteria used in different studies. The higher prevalence found in this review could be attributed to several factors, including variations in trauma exposure, differences in diagnostic criteria (e.g., DSM-IV vs. DSM-5), the subjective nature of PTSD diagnoses, or differences in population characteristics such as age, gender, and cultural background [12, 35].

The implications of these findings are considerable. If the prevalence of PTSD among children and adolescents is indeed higher than previously documented, there is a pressing need for enhanced screening, early intervention, and targeted support services that are specifically designed to address the unique needs of children and adolescents. Furthermore, this may indicate a gap in current understanding and awareness of trauma’s long-term effects on youth, necessitating further research to explore the underlying causes of this elevated prevalence, including longitudinal studies to track the progression of PTSD symptoms over time and studies that examine the effectiveness of different intervention strategies. Addressing these issues could ultimately lead to improved mental health outcomes for children and adolescents affected by trauma.

The exceptional degree of heterogeneity (I² = 99.9%) suggests that the studies included in your review are highly diverse. This could be due to various factors such as differences in the types of trauma experienced, the age of the children, cultural contexts, and diagnostic criteria used [36]. High heterogeneity can complicate the interpretation of pooled estimates and suggests that subgroup analyses might be necessary to understand the sources of variability better.

The observed heterogeneity in our umbrella review prompted us to conduct subgroup analyses to better understand the variability in PTSD prevalence among children and adolescents. By analyzing the data based on the year of publication and the number of primary studies included in the SRMAs, we aimed to identify potential sources of this heterogeneity and provide a more nuanced understanding of PTSD prevalence.

Our subgroup analysis revealed notable differences in prevalence rates based on the publication year of the SRMAs. It disclosed a concerning increase in the prevalence of PTSD among children and adolescents, with rates rising from 17% in SRMAs published more than five years ago to 28% in those published within the past five years. This significant increase warrants a closer examination of the factors contributing to this trend. One potential explanation for the rising prevalence is the increased awareness and recognition of PTSD as a critical mental health issue affecting children and adolescents. Over recent years, there has been a concerted effort to improve screening practices and diagnostic capabilities, leading to better identification of PTSD cases. This heightened awareness among clinicians and researchers may contribute to the observed increase in reported prevalence rates, as more individuals are diagnosed and treated for PTSD than in previous years [37]. Additionally, the growing exposure to traumatic events among children and adolescents cannot be overlooked. Factors such as natural disasters, armed conflicts, and societal unrest have become more prevalent, potentially leading to higher rates of trauma exposure [38]. For instance, the COVID-19 pandemic introduced new stressors that have significantly impacted the mental health of young people, further contributing to the rise in PTSD prevalence [20, 23]. Moreover, methodological advancements in recent studies may also play a role in the increased prevalence rates. Recent SRMAs often employ more rigorous methodologies, including standardized diagnostic criteria and comprehensive data collection techniques, which can yield more accurate prevalence estimates. This methodological rigor may explain the higher rates reported in more recent studies compared to those conducted in the past [39].

The subgroup analysis also indicated variations based on the number of primary studies included in the SRMAs. For SRMAs that assessed PTSD using fewer than 20 primary studies, the prevalence was 24% (95% CI: 16–32%) with an I² of 99.9%, compared to 28% (95% CI: 19–36%) for those including 20 or more studies (I² = 99.8%). The higher prevalence in SRMAs with more studies may reflect a broader range of contexts and trauma exposures, leading to a more representative estimate of the prevalence of PTSD. Larger sample sizes tend to provide more robust data, allowing for better generalizability of findings, as supported by the literature indicating that larger studies often yield more reliable prevalence estimates due to increased statistical power and diversity of trauma experiences captured [39, 40]. However, the consistently high I² values across both categories indicate substantial variability, suggesting that even with larger samples, factors such as study design, population characteristics, and types of trauma may still influence reported prevalence rates. This variability underscores the complexity of PTSD epidemiology, where different methodologies and contextual factors can lead to divergent findings, as noted in previous reviews [12]. Therefore, while the overlap in confidence intervals between the two groups suggests that the differences in prevalence may not be statistically significant, it is essential to consider the broader implications of these findings in understanding the nuances of PTSD prevalence among children and adolescents.

The development of PTSD among children and adolescents is influenced by a complex interplay of risk factors. Research consistently indicates that older age is a significant risk factor for PTSD [36, 41, 42]. Adolescents, particularly those over 14 years, are more likely to develop PTSD following traumatic experiences compared to younger children. This increased vulnerability may be attributed to greater cognitive and emotional awareness, which can intensify the psychological impact of trauma [9, 11, 12]. As children mature, they may also have more complex emotional responses and a deeper understanding of the implications of traumatic events, which can exacerbate feelings of fear and helplessness [42, 43].

In addition, gender differences play a critical role in the development of PTSD. Studies have shown that females are at a higher risk for developing PTSD than males, with prevalence rates often reported to be twice as high in girls [30–33, 44–48]. This disparity may be influenced by several factors, including differences in exposure to trauma, societal expectations, and coping mechanisms. Girls are more likely to experience certain types of trauma, such as sexual abuse, which are strongly associated with PTSD [49]. Additionally, females may be more likely to internalize their emotions, leading to a higher incidence of PTSD symptoms.

Moreover, the presence or absence of social support is a critical factor in the development of PTSD. Low social support has been consistently associated with higher rates of PTSD among children and adolescents [9, 11]. Supportive relationships with family, peers, and community members can act as protective factors, helping children process traumatic experiences and mitigate the psychological impact [50]. Conversely, children who lack supportive networks may feel isolated and unable to cope with their trauma, increasing their risk for PTSD [50].

Experiencing feelings of entrapment, fear, or helplessness during a traumatic event has also been identified as a significant risk factor for PTSD [11, 49]. These emotions can intensify the psychological impact of trauma and hinder recovery. Children who perceive themselves as powerless in the face of trauma may struggle to regain a sense of control, which is essential for healing. Interventions that focus on empowering children and fostering resilience can be beneficial in reducing the risk of PTSD [22].

Another significant factor, experiencing bereavement, particularly the death of a family member or loved one, is a significant risk factor for PTSD in children and adolescents [11, 12]. The emotional turmoil associated with loss can lead to profound grief and complicate the trauma recovery process [9]. Children who witness the death of others, especially in violent or traumatic circumstances, are also at heightened risk for developing PTSD. This underscores the need for targeted interventions that address grief and loss in therapeutic settings.

The interplay of these risk factors emphasizes the need for comprehensive approaches to prevention and intervention for PTSD in children and adolescents. By addressing age, gender differences, social support, emotional responses, and bereavement experiences, practitioners can better support this vulnerable population in their recovery journeys.

This umbrella review has several limitations that should be acknowledged. Firstly, the included studies exhibit substantial heterogeneity (I² = 99.9%), which may affect the reliability of the pooled prevalence estimates and risk factor analysis. Despite efforts to minimize bias, the possibility of publication bias remains, as studies with significant findings are more likely to be published. Additionally, the review identified only two systematic reviews and meta-analyses specifically addressing risk factors, limiting the comprehensiveness of the risk factor analysis. Differences in diagnostic criteria and assessment tools across studies may contribute to variability in reported prevalence rates. The studies included span a decade and various geographical regions, which may introduce temporal and cultural biases affecting the generalizability of the findings. The quality of the included studies ranged from low to moderate, which may impact the robustness of the conclusions drawn. Furthermore, the review included only studies published in English and within a specific timeframe (2014–2024), potentially excluding relevant research published in other languages or outside this period. These limitations should be acknowledged to provide a balanced interpretation of the findings and to guide future research in this area.

### Study implications

The high prevalence of PTSD among children and adolescents exposed to trauma necessitates significant clinical and policy interventions. Clinically, it is crucial to implement routine screening for PTSD in pediatric healthcare settings, particularly for those who have experienced trauma. Early identification can lead to timely and effective interventions. Developing targeted therapeutic interventions that address specific risk factors such as older age, female gender, and low social support can enhance treatment outcomes. Integrated care models that combine mental health services with primary care should be promoted to ensure comprehensive care addressing both physical and psychological needs. Additionally, specialized training for healthcare providers on recognizing and managing PTSD in younger populations is essential. Family involvement in the treatment process should be encouraged, as family support plays a critical role in the recovery of children and adolescents with PTSD.

From a policy perspective, there is a need for increased funding for mental health services aimed specifically at children and adolescents. This includes funding for research, prevention programs, and treatment facilities. Implementing and expanding school-based mental health programs can provide crucial support and resources for students affected by trauma. Public awareness campaigns are necessary to educate communities about the prevalence and impact of PTSD in children and adolescents, reducing stigma and encouraging families to seek help. Developing and enforcing policies that protect children from traumatic experiences, such as abuse and violence, is also vital. This includes strengthening child protection services and ensuring safe environments for children. Supporting ongoing research and data collection on PTSD in children and adolescents will inform evidence-based policy decisions, including longitudinal studies to track the long-term effects of trauma and the effectiveness of interventions.

### Future research directions

The scarcity of high-quality, aggregated data on the determinants of PTSD in children and adolescents has underscored a critical gap in the existing literature. This review has highlighted the need for further systematic reviews and meta-analyses to better understand the multifaceted nature of PTSD risk factors in this population. Addressing these gaps will enable future research to contribute to the development of more effective prevention and intervention strategies, tailored to the unique needs of children and adolescents affected by PTSD.

## Conclusion

The prevalence of PTSD among children and adolescents exposed to trauma is notably high. This significant finding underscores the profound impact of traumatic experiences on young individuals. Key contributing factors include older age, female gender, low social support, feelings of entrapment, and experiencing bereavement. To address these issues, it is crucial to implement targeted interventions that focus on these risk factors. Enhancing social support systems, providing early and effective mental health interventions, and developing tailored therapeutic approaches can significantly improve outcomes for children and adolescents affected by trauma. Further research is essential to refine these strategies and ensure they are effectively meeting the needs of this vulnerable population.

## Electronic supplementary material

Below is the link to the electronic supplementary material.

Supplementary Material 1.

Supplementary Material 2.

## Data Availability

Data analyzed during preparation of this study has been submitted as a supplimentary file.

## References

[1] Bakker L-P. A follow-up study after the Vassdalen avalanche: Surviving soldiers’ self-report and experiences 30 years post-disaster. 2020.

[2] Regier DA, Kuhl EA, Kupfer DJ. The DSM-5: classification and criteria changes. World Psychiatry. 2013;12(2):92–8. 23737408 10.1002/wps.20050 PMC3683251

[3] Keller SM, Burton M, Feeny NC. Posttraumatic stress disorder. Clinical handbook of psychological disorders in children and adolescents: A step-by-step treatment manual. 2017:240– 72.

[4] Meiser-Stedman R. Q11 Q12 systematic review and Meta-analysis: Prevalence of Posttraumatic Stress Disorder in Trauma-Exposed Preschool-Aged Children. 10.1016/j.jaac.2021.05.026 PMC8885427 34242737

[5] Atwoli L, Stein DJ, Koenen KC, McLaughlin KAJC. Epidemiology of posttraumatic stress disorder: prevalence, correlates and consequences. 2015;28(4):307–11. 10.1097/YCO.0000000000000167 PMC4452282 26001922

[6] Mobbs MC, Bonanno GAJC. Beyond war and PTSD: the crucial role of transition stress in the lives of military veterans. 2018;59:137–44. 10.1016/j.cpr.2017.11.007 29180101

[7] Monson CM, Taft CT, Fredman, SJJCpr. Military-related PTSD and intimate relationships: from description to theory-driven research and intervention development. 2009;29(8):707–14. 10.1016/j.cpr.2009.09.002 PMC2783889 19781836

[8] DiMauro J, Carter S, Folk JB, Kashdan, TBJJoad. A historical review of trauma-related diagnoses to reconsider the heterogeneity of PTSD. 2014;28(8):774–86. 10.1016/j.janxdis.2014.09.002 25261838

[9] Hamblen J, Barnett EJBM. PTSD: National center for PTSD. 2018:366-7.

[10] McLaughlin KJUe. Posttraumatic stress disorder in children and adolescents: Epidemiology, clinical features, assessment, and diagnosis. 2023.

[11] Tang B, Deng Q, Glik D, Dong J, Zhang LJI. health p. A meta-analysis of risk factors for post-traumatic stress disorder (PTSD) in adults and children after earthquakes. 2017;14(12):1537. 10.3390/ijerph14121537 PMC5750955 29292778

[12] Tamir TT, Yimer B, Gezahgn SA, Mekonnen FA, Teshome DF, Angaw DAJB. Prevalence and associated factors of post-traumatic stress disorder in pediatric populations in Africa: a systematic review and meta-analysis. 2024;24(1):643. 10.1186/s12888-024-06106-2 PMC11443807 39350116

[13] Tortella-Feliu M, Fullana MA, Pérez-Vigil A, Torres X, Chamorro J, Littarelli SA, et al. Risk factors for posttraumatic stress disorder: an umbrella review of systematic reviews and meta-analyses. Neurosci Biobehavioral Reviews. 2019;107:154–65. 10.1016/j.neubiorev.2019.09.013 31520677

[14] Tian Y, Wong TK, Li J, Jiang X. Posttraumatic stress disorder and its risk factors among adolescent survivors three years after an 8.0 magnitude earthquake in China. BMC Public Health. 2014;14:1–7. 25318533 10.1186/1471-2458-14-1073 PMC4210499

[15] Salmon K, Bryant RAJC. Posttraumatic stress disorder in children: the influence of developmental factors. 2002;22(2):163–88. 10.1016/s0272-7358(01)00086-1 11806018

[16] Li Y, Zhou Y, Chen X, Fan F, Musa G, Hoven CJPm. Post-traumatic stress disorder in children and adolescents: Some recent research findings. 2020.

[17] Downey C, Crummy AJEJoT. Dissociation. The impact of childhood trauma on children’s wellbeing and adult behavior. 2022;6(1):100237.

[18] Shea BJ, Reeves BC, Wells G, Thuku M, Hamel C, Moran J et al. AMSTAR 2: a critical appraisal tool for systematic reviews that include randomised or non-randomised studies of healthcare interventions. Or both. 2017;358. 10.1136/bmj.j4008 PMC5833365 28935701

[19] Ioannidis JP. Interpretation of tests of heterogeneity and bias in meta-analysis. J Eval Clin Pract. 2008;14(5):951–7. 19018930 10.1111/j.1365-2753.2008.00986.x

[20] Alizadeh S, Shahrousvand S, Sepandi M, Alimohamadi YJJPH. Prevalence of anxiety, depression and post-traumatic stress disorder symptoms in children and adolescents during the COVID-19 pandemic: A systematic review and meta-analysis. 2023:1–16.

[21] Ozamiz-Etxebarria N, Legorburu Fernandez I, Idoiaga-Mondragon N, Olaya B, Cornelius-White JH, Santabárbara JJS. Post-traumatic stress in children and adolescents during the COVID-19 pandemic: A meta-analysis and intervention approaches to ensure mental health and well-being. 2023;15(6):5272.

[22] Kanan J, Leão T. Post-traumatic stress disorder in youth exposed to the Syrian conflict: A systematic review and meta-analysis of prevalence and determinants. J Health Psychol. 2022;29(13):1433–49. 36124723 10.1177/13591053221123141 PMC11538769

[23] Yang F, Wen J, Huang N, Riem MM, Lodder P, Guo JJE. Prevalence and related factors of child posttraumatic stress disorder during COVID-19 pandemic: A systematic review and meta-analysis. 2022;65(1):e37. 10.1192/j.eurpsy.2022.31 PMC9280924 35726735

[24] Agbaria N, Petzold S, Deckert A, Henschke N, Veronese G, Dambach P et al. Prevalence of post-traumatic stress disorder among Palestinian children and adolescents exposed to political violence: A systematic review and meta-analysis. 2021;16(8):e0256426. 10.1371/journal.pone.0256426 PMC8389374 34437595

[25] Woolgar F, Garfield H, Dalgleish T, Meiser-Stedman RJJotAAoC, Psychiatry A. Systematic review and meta-analysis: prevalence of posttraumatic stress disorder in trauma-exposed preschool-aged children. 2022;61(3):366–77. 10.1016/j.jaac.2021.05.026 PMC8885427 34242737

[26] Turgoose DP, Kerr S, De Coppi P, Blackburn S, Wilkinson S, Rooney N et al. Prevalence of traumatic psychological stress reactions in children and parents following paediatric surgery: a systematic review and meta-analysis. 2021;5(1). 10.1136/bmjpo-2021-001147 PMC8287603 34337164

[27] Latuperissa GR, Rumaolat W, Susanti I, Soulisa FFJJN. A systematic review of the effect of social support on Post-Traumatic stress disorder in Post-Earthquake adolescents. 2020;15(2).

[28] Dai W, Liu A, Kaminga AC, Deng J, Lai Z, Wen SWJTCJP. Prevalence of posttraumatic stress disorder among children and adolescents following road traffic accidents: a meta-analysis. 2018;63(12):798–808. 10.1177/0706743718792194 PMC6309043 30081648

[29] Alisic E, Zalta AK, Van Wesel F, Larsen SE, Hafstad GS, Hassanpour K et al. Rates of post-traumatic stress disorder in trauma-exposed children and adolescents: meta-analysis. 2014;204(5):335–40. 10.1192/bjp.bp.113.131227 24785767

[30] Mbwayo AW, Mathai M, Harder VS, Nicodimos S, Vander Stoep A. Trauma among Kenyan school children in urban and rural settings: PTSD prevalence and correlates. J Child Adolesc Trauma. 2020;13:63–73. 32318229 10.1007/s40653-019-00256-2 PMC7163810

[31] Arega NT. Posttraumatic stress among Eritrean unaccompanied refugee minors in Ethiopia. Int J Migration Health Social Care. 2020;17(1):1–15.

[32] Astitene K, Barkat A. Prevalence of posttraumatic stress disorder among adolescents in school and its impact on their well-being: a cross-sectional study. Pan Afr Med J. 2021;39(1). 10.11604/pamj.2021.39.54.27419 PMC8363972 34422177

[33] Tamir TT, Kassa SF, Gebeyehu DA. A multi-institutional study of post-traumatic stress disorder and its risk factors in Ethiopian pediatric patients with physical trauma. BMC Psychiatry. 2022;22(1):271. 35428231 10.1186/s12888-022-03930-2 PMC9011951

[34] Corales TA. Trends in posttraumatic stress disorder research. Nova; 2005.

[35] Diagnostic. and statistical manual of mental disorders: DSM-5™, 5th ed, (2013). 10.1590/s2317-17822013000200017 24413388

[36] Stupar D, Stevanovic D, Vostanis P, Atilola O, Moreira P, Dodig-Curkovic K, et al. Posttraumatic stress disorder symptoms among trauma-exposed adolescents from low- and middle-income countries. Child Adolesc Psychiatry Mental Health. 2021;15(1):26. 10.1186/s13034-021-00378-2 PMC8180049 34090487

[37] Astitene K, Barkat AJPAMJ. Prevalence of posttraumatic stress disorder among adolescents in school and its impact on their well-being: a cross-sectional study. 2021;39(1). 10.11604/pamj.2021.39.54.27419 PMC8363972 34422177

[38] Scheeringa MS, Zeanah CH, Cohen JAJD, editors. anxiety. PTSD in children and adolescents: toward an empirically based algorithm a. 2011;28(9):770– 82. 10.1002/da.20736 PMC6101653 20734362

[39] Schincariol A, Orrù G, Otgaar H, Sartori G, Scarpazza CJPM. Posttraumatic stress disorder (PTSD) prevalence: an umbrella review. 2024:1–14. 10.1017/S0033291724002319 PMC11650175 39324396

[40] Tortella-Feliu M, Fullana MA, Pérez-Vigil A, Torres X, Chamorro J, Littarelli SA et al. Risk factors for posttraumatic stress disorder: an umbrella review of systematic reviews and meta-analyses. 2019;107:154–65. 10.1016/j.neubiorev.2019.09.013 31520677

[41] Nilaweera D, Phyo AZZ, Teshale AB, Htun HL, Wrigglesworth J, Gurvich C, et al. Lifetime posttraumatic stress disorder as a predictor of mortality: a systematic review and meta-analysis. BMC Psychiatry. 2023;23(1):229. 37032341 10.1186/s12888-023-04716-w PMC10084620

[42] Pate KM. Understanding Post-Traumatic stress disorder in children: A comprehensive review. Inquiries J. 2021;13(02).

[43] Hamblen J, Barnett E. PTSD in children and adolescents. National Center for PTSD website; 2016.

[44] Shekwolo DM, Monday A, Sule OS, Temidayo OF, AsheaziAugustine D. Posttraumatic stress disorder symptoms among women and children affected by Boko Haram insurgency in North-east Nigeria. Nigerian J Psychol Res. 2017;13.

[45] Astitene K, Aguenaou H, Lahlou L, Barkat A. Prevalence of post-traumatic stress disorder among school-age adolescent. Int Neuropsychiatr Dis J. 2020;14:40–9.

[46] Ainamani HE, Weierstall-Pust R, Bahati R, Otwine A, Tumwesigire S, Rukundo GZ. Post-traumatic stress disorder, depression and the associated factors among children and adolescents with a history of maltreatment in Uganda. Eur J Psychotraumatology. 2022;13(1):2007730. 10.1080/20008198.2021.2007730 PMC8751492 35028113

[47] Boudabous J, Kerkeni A, Kraiem M, Ayadi H, Moalla Y. Post-traumatic stress disorder in adolescents during the COVID-19 pandemic: a cross-sectional Tunisian study. Middle East Curr Psychiatry. 2023;30(1):101.

[48] Awad MH, Abbas MM, Mohamed RS, Mohamed HO. Posttraumatic stress disorder: point prevalence and risk factors among Sudanese children and adolescents during Sudan army conflict. A Cross Sectional Study; 2024. 10.1007/s44192-024-00084-3 PMC11327231 39145898

[49] Trickey D, Siddaway AP, Meiser-Stedman R, Serpell L, Field AP. A meta-analysis of risk factors for post-traumatic stress disorder in children and adolescents. Clin Psychol Rev. 2012;32(2):122–38. 22245560 10.1016/j.cpr.2011.12.001

[50] Xiong T, Milios A, McGrath PJ, Kaltenbach EJEJP. The influence of social support on posttraumatic stress symptoms among children and adolescents: a scoping review and meta-analysis. 2022;13(1):2011601. 10.1080/20008198.2021.2011601 PMC8942489 35340789

